# Comparison of MEMS switches and PIN diodes for switched dual tuned RF coils

**DOI:** 10.1002/mrm.27156

**Published:** 2018-03-09

**Authors:** Adam Maunder, Madhwesha Rao, Fraser Robb, Jim M. Wild

**Affiliations:** ^1^ Unit of Academic Radiology, University of Sheffield United Kingdom; ^2^ GE Healthcare Aurora Ohio

**Keywords:** Micro‐electromechanical systems (MEMS), PIN diode, fluorine‐19 MRI, Switchable RF coils, Dual Tuned RF coils, lung MRI

## Abstract

**Purpose:**

To evaluate the performance of micro‐electromechanical systems (MEMS) switches against PIN diodes for switching a dual‐tuned RF coil between ^19^F and ^1^H resonant frequencies for multi‐nuclear lung imaging.

**Methods:**

A four‐element fixed‐phase and amplitude transmit–receive RF coil was constructed to provide homogeneous excitation across the lungs, and to serve as a test system for various switching methods. The MR imaging and RF performance of the coil when switched between the ^19^F and ^1^H frequencies using MEMS switches, PIN diodes and hardwired configurations were compared.

**Results:**

The performance of the coil with MEMS or PIN diode switching was comparable in terms of RF measurements, transmit efficiency and image SNR on both ^19^F and ^1^H nuclei. When the coil was not switched to the resonance frequency of the respective nucleus being imaged, reductions in the transmit efficiency were observed of 32% at the ^19^F frequency and 12% at the ^1^H frequency. The coil provides transmit field homogeneity of ±12.9% at the ^1^H frequency and ±14.4% at the ^19^F frequency in phantoms representing the thorax with the air space of the lungs filled with perfluoropropane gas.

**Conclusion:**

MEMS and PIN diodes were found to provide comparable performance in on‐state configuration, while MEMS were more robust in off‐state high‐powered operation (>1 kW), providing higher isolation and requiring a lower DC switching voltage than is needed for reverse biasing of PIN diodes. In addition, clear benefits of switching between the ^19^F and ^1^H resonances were demonstrated, despite the proximity of their Larmor frequencies.

## 1 INTRODUCTION

In non‐proton MRI applications, it is desirable to be able to acquire ^1^H structural imaging that is co‐registered to the complementary functional imaging provided by the other nucleus, as demonstrated previously with hyperpolarized gas and ^1^H lung MRI.^1^ The motivation for this work was development of switched dual‐tuned RF coil designs to allow detection of inhaled ^19^F C_3_F_8_ gas and ^1^H signals from the lungs at 1.5 T in the same scan session.

In previous human lung imaging studies with perfluorinated ^19^F gases, the ^1^H body coil has typically been used with an actively decoupled ^19^F vest coil.^2^ The use of a coil for both ^1^H and ^19^F nuclei without dual‐tuning has been implemented previously,^3^ but the detection sensitivity and homogeneity was only optimized at the ^19^F frequency. Trap circuits are commonly used to tune the coil resonance to multiple frequencies^4^ using inductive and capacitive elements in parallel. However, for ^19^F (60.06 MHz at 1.5 T) and ^1^H (63.8 MHz at 1.5 T) the bandwidth of passive traps with the typical Q‐factors of commercially available components is comparable to the frequency separation, limiting their use, as discussed previously.^5^ Another approach is to actively switch‐in capacitors in parallel to the existing tuning capacitors using PIN diodes, and more recently the use of field effect transistors^6^ and micro‐electromechanical systems (MEMS) 7, 8, 9 have also been reported. The equivalent series resistance (ESR) of these three devices are reported to be insignificant when compared with the quality factor ( Q) of trap circuit inductors (eg. Q∼ 120 at 128 MHz in Maunder et al),^10^ which results in negligible additional loss. For example, when comparing a dual‐tuned coil design to single tuned counterparts, SNR losses of 25% and 50% were reported for ^19^F and ^1^H, respectively,^11^ while the switching used in Choi et al^12^ resulted in more equivalent performance for imaging both ^19^F and ^1^H when compared with respective single tuned coils. Therefore, due to the close frequencies of ^19^F and ^1^H at 1.5 T the use of switching is favored.

Recent improvements in the technology for MEMS switches and associated driver circuitry has allowed increased switching speed, better power handling and reduced insertion loss,^13^ so that MEMS switches have been successfully demonstrated for coil decoupling^14^ and reconfigurable RF coils^15^ in MRI. However, MEMS are not well established or characterized for use in the MRI environment when compared with PIN diodes, so both devices are systematically compared here. A summary of typical performance parameters for FETs, MEMS or PIN diodes is presented in Supporting Information Table S1, which is available online, with the specific values for the PIN diodes and MEMS components used in this study. Notably, the switching speed has typically been found to be limited by the driver circuitry rather than the devices themselves.^6^ It would, therefore, be beneficial to use low DC power MEMS or FETs for switching, but for FETs the breakdown voltage is lower than often present for high power transmission pulses, which restricts their use in MRI.

In this Note, we compare two methods for switching the matching network tuning: MEMS and PIN diodes, and compare these with a hard‐wired configuration for either nucleus. The switching comparison is exemplified using a four element fixed phase/amplitude transmit–receive RF coil designed for lung imaging of ^1^H and ^19^F perfluorinated gases at 1.5 T.

## 2 METHODS

### 2.1 Component evaluation: Power handling of PIN diode and MEMS

**Figure 1:**
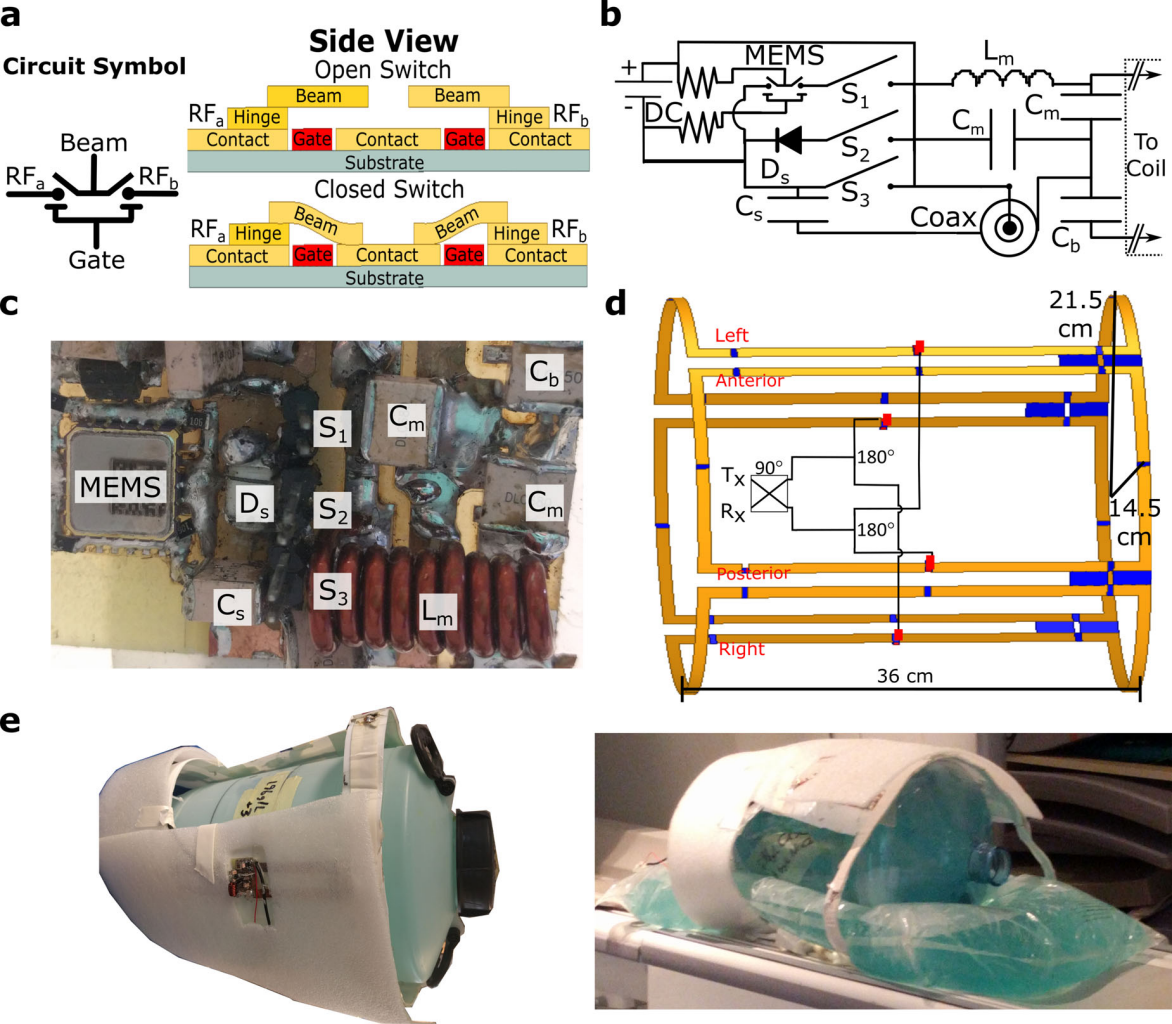
(**a**) Circuit schematic of MEMS switch used here and wafer level representative diagrams of the devices used. (**b**) A circuit schematic of the matching network design using the various switching methods. (**c**) The constructed matching network on anterior coil. (**d**) Schematic of transmit‐receive coil for ^19^F and ^1^H imaging at 1.5 T with dimensions labeled. Included in the driving circuitry is a 90 ° hybrid, a pair of 180 ° splitters/combiners, and a T/R switch. (**e**) The coil prototype with cylindrical and bag phantom used to emulate body loading

The mechanism of actuating MEMS switches is fundamentally different to that of PIN diodes. The MEMS used here consist of an array of beam type structures that operate as relays actuated electrostatically by a DC voltage applied between the beam and gate.^14^ A representative side view of the MEMS structure is shown in Figure 1A displaying the method of operation, as the switch is actuated the beams make contact with the central conductor providing a connection between RF_a_ and RF_b_ in the circuit schematic model. More details on the device structure are provided in Keimel et al.^13^ To compare the behavior of both PIN diode and MEMS under the higher RF transmit power conditions experienced in whole‐body MRI, a bench‐top test was set up. A pulse‐modulated signal of 60 MHz with pulse duration of 0.2 ms (duty cycle 0.02%) was generated by a WS8352‐Taber waveform generator. A 335953‐Picker linear pulse amplifier was used to generate peak output powers from 7.3–2380 W. The output time‐domain voltage waveform was measured on a high‐speed oscilloscope (DSO 104A‐Keysight) after 30 dB attenuation. Transmission to the attenuator was through MEMS switch or PIN diode placed in series, and DC bias isolated by choke inductors. The MEMS switch configuration was evaluated in open or closed position, and the PIN diode configuration was evaluated with varying reverse bias voltages and forward bias currents.

### 2.2 Coil design for switching application

To test the switching performance, a four element fixed phased transmit–receive coil was designed for dual tuned use for imaging ^19^F (60.06 MHz) and ^1^H (63.8 MHz) at 1.5 T on a GE Signa HDx system equipped with a 4 kW broadband RF amplifier. A circularly polarized $B_{1}^{+}$ excitation was achieved using a combination of 90° and 180° hybrid circuits that were custom built for both the ^1^H and ^19^F frequencies. In the matching network topography shown in Figure 1B, the capacitor C_s_ was switched‐in to change the matching tuning (resonance) of the coil from the Larmor frequency of ^1^H to ^19^F by three possible mechanisms:

(i) MEMS (MM7100, MenloMicro, Irvine, CA) switched on by application of 82 V DC,

(ii) PIN diode (MA4P7435F‐1091T, MACOM, MA) forward biased with 100 mA DC current,

(iii) Hard‐wired configuration for either nucleus.

The matching capacitance (10C package, Dali Capacitors, Dalian, China) and inductance values were; $C_{m}=68\text{ pF }$ and $L_{m}=92\;nH$ for the left and right coils and $C_{m}=56$ pF and $L_{m}=111$ nH for the anterior and posterior coils, as annotated in Figure 1C. The capacitor was $C_{s}=75$ pF for all four coil elements. The manufactured matching networks are shown in Figure 1C. RF scattering parameters were measured on the bench using an Agilent E5061A Network Analyzer (Keysights, Santa Clara, CA). To characterize the loss of the matching networks, the scattering parameters of the matching network were measured without the coil connected and the power loss ratio, $P_{LR}$, was calculated.^16^

Decoupling between adjacent coil elements was achieved using capacitive decoupling networks.^17^ The topology and dimensions of the coil (Figure 1D) were designed to provide a receive sensitivity and transmit field profile that covered the lungs of a large adult male, with <20% variation over a 25 × 25 × 20 cm^3^ volume. The widths were 25.5 cm for the anterior/posterior and 30 cm for the right/left elements coils. The coils were constructed from 11‐mm‐wide and 77‐ μm‐thick self‐adhesive copper tape mounted on a flexible Polytetrafluoroethylene substrate. There were five capacitor break‐points in each coil.

For ^1^H imaging and RF measurements a cylindrical phantom was used consisting of 3.6 g/L NaCl and 1.96 g/L CuSO_4_⋅5H_2_O salt solution^18^ to represent a human load. For ^19^F imaging two glass canisters (2 L volume) were filled with C_3_F_8_ gas mixed with 21% O_2_ at 1.5 bar pressure, which emulates the air‐space in the human thorax. The glass phantoms were placed in a cylindrical shell and surrounded with a 12 L bag containing the saline solution and placed over another equal volume bag for suitable loading. The phantoms and coil are shown in Figure 1E.

### 2.3 Imaging tests

Measurement of T_1_ was performed in homogeneous phantoms by 2D spoiled gradient echo (SPGR) imaging. First, the flip angle (FA) was fit against image intensity with varying input power with TR >>T_1_ (TR = 600 ms for ^1^H phantom experiments and TR = 80 ms for ^19^F in the C_3_F_8_/O_2_ phantom). Next, T_1_ was fit against the image intensity with varying FA but with TR<T_1_ (TR = 8 ms for ^1^H phantom experiments and TR = 7.5 ms for ^19^F in C_3_F_8_/O_2_ phantom). Image intensity ( $S_{SPGR})$ was related to FA and $T_{1}$ according to Deoni^19^

(1) $$S_{SPGR}=\frac{\rho \left(1-e^{-TR/T_{1}}\right)\sin FA}{\sigma \left(1-e^{-\frac{TR}{T_{1}}}\cos FA\right)},$$

where ρ is a proportionality factor that depends upon system hardware and the TE, which was fixed. The noise standard deviation, σ, is included to normalize the signal to SNR units. The standard deviation of noise in images was measured in a signal‐free region of greater than 100 pixels as described in NEMA.^20^

To compare the effect of the respective switched tuning methods on transmit efficiency, the FAs were measured for ^1^H and ^19^F SPGR imaging by varying input power and fitting FA, as well as ρ/σ, according to Eq. ((1)) with the phantom measured T_1_'s for ^1^H and ^19^F (reported in results section) when the coil tuning was set to both ^19^F and ^1^H, respectively. The corresponding transmit efficiency with known input power and pulse width was subsequently calculated.

### 2.4 In vivo imaging

In vivo lung imaging evaluation was performed with inhaled C_3_F_8_ mixed with 21% O_2_ with a healthy adult male volunteer (28 years) following informed consent and a protocol approved by UK National research ethics committee. Three‐breaths of the gas were inhaled and then 3D ^19^F SPGR imaging was performed within a single breath‐hold (37 s scan time). In addition, ^1^H 3D SPGR anatomical imaging was performed during a separate breath‐hold (13 s) of air with the lungs at the same inflation level. Both images were localized to cover the same geometry. MEMS were used to switch between the two tuning states during in vivo imaging. A summary of all sequence and acquisition parameters used for the imaging experiments are provided in Table 1.

**Table 1:** Imaging parameters for coil performance evaluation

Measurement	Sequence	TE (ms)	TR (ms)	BW ( ± kHz)	Matrix size	FOV (cm^3^)	Mean FA (°)	Avg.
^1^H – T_1_	2D Axial SPGR	4.6	FA fit ‐ 600 T_1_ fit ‐ 8	14.76	128 × 128 × 1	40 × 40 × 1	varied	1
^19^F – T_1_	2D Axial SPGR	3.4	FA fit ‐ 80 T_1_ fit ‐ 7.5	8.06	30 × 25 × 1	30 × 24 × 10	‐	20
^1^H – Tx efficiency	3D Coronal SPGR	3.1	8 ms	31.25	128 × 96 × 30	44 × 33 × 30	‐	1
^19^F – Tx efficiency	3D Coronal SPGR	2.1	5.1	6.94	50 × 42 × 10	30 × 24 × 20	‐	10
^1^H – In‐Vivo	3D Coronal SPGR	3.7	9.1	8.06	100 × 100 × 28	42 × 42 × 28	35	1
^19^F – In‐Vivo	3D Coronal SPGR	0.9	4.3	10	50 × 42 × 14 (75% kx)	42 × 34 × 28	27	15

## 3 RESULTS

### 3.1 Coil bench testing

**Figure 2:**
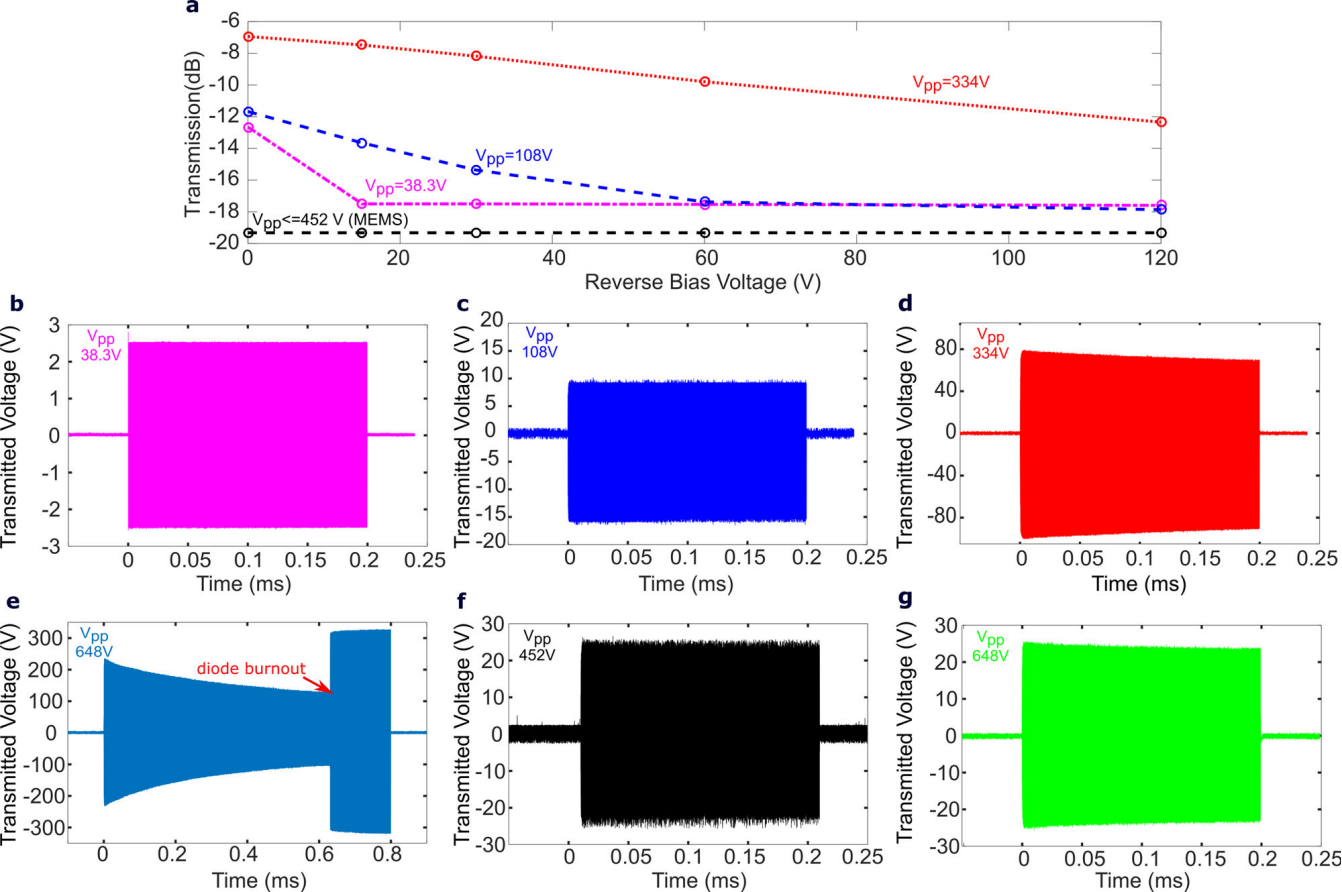
(**a**) Transmission relative to operation in the conducting state for MEMS and PIN diode with different reverse bias voltages. The measurement waveforms with increasing RF power are displayed below. The V_pp_ labeled next to waveforms is the voltage measured in the forward conducting state with 100 mA bias current. Waveforms are shown for when PIN diode is reverse biased by 15V at **b** (Vpp = 38.3 V), **c** (Vpp = 108 V), and **d** (Vpp = 334 V). (**e**) The waveform for the PIN diode during an extended high‐power RF pulse shows voltage droop leading to failure, which is not evident for either F (MEMS in open‐state), or **g** (a series 3 pF blocking capacitor)

Both the MEMS when switched closed, and PIN diode when forward biased, remained operational up to the maximum powers tested (2380 W, or approximately 6.9 A peak current), in accordance with the maximum values specified in Supporting Information Table S1. However, as demonstrated in Figure 2A, the reverse biased PIN diode began to conduct RF power when the reverse bias DC voltage was lower than the peak RF voltage, which was not the case with the MEMS switch in the open position. However, at the maximum power (equivalent to a peak voltage of 690 Vpp as delivered to a 50 Ω load), the MEMS switch in the open position suffered critical failure. Figure 2B–D displays the measured pulse waveforms with increasing RF power and 15 V reverse bias of the PIN diode demonstrating conduction was primarily coming from undesired injection of majority carriers in the intrinsic region on the negative voltage swing.

Additionally, at high power there was an observed droop in the voltage over the pulse length when the diode was insufficiently reverse biased. This is believed to be related to rapid heating of the PIN diode during the RF pulse resulting in increased impedance. The waveform observed (Figure 2D) when the pulse length and power was increased (Vpp = 648V) shows the effect became more pronounced and ultimately led to device failure. This voltage droop was only observed for PIN diodes when insufficiently reverse biased, the waveforms obtained at the same power with either MEMS or a 3 pF blocking capacitor (Figure 2F,G) showed no droop. The MEMS switch used here was found to have a marginally higher isolation (19.4 dB) when compared with the diode (18 dB) when a sufficiently high reverse bias voltage was applied. The power levels tested on the bench were higher than those expected in the scanner and with the maximum 1 kW RMS input pulse there was no observed unintentional reverse biasing of the forward‐biased PIN diode or failure of the MEMS switch.

The measured unloaded and loaded quality factors of coils were 165 and 14.4 for anterior/posterior coils and 195 and 14.3 for right/left coils, respectively. The measured coil resistance when loaded with a cylindrical phantom was ∼24 Ω for the anterior/posterior coils and ∼26 Ω for left/right coils (Figure 1E). The reflection coefficient of one of the coil elements (right) when switched between the ^19^F and ^1^H frequencies by means of each of the three methods is shown in Supporting Information Figure S1. The reflection coefficients of all the elements were found to be less than ‐20 dB at the frequencies of interest (60.06 MHz for ^19^F and 63.8 MHz for ^1^H). The 90 ° hybrid and 180 ° power dividers used had a reflection coefficient less than ‐15 dB for both frequencies with insertion loss of ∼0.5 dB. The decoupling between nearest neighbor coils (e.g., anterior and right) was optimized for the ^19^F frequency, where isolation was greater than 15 dB for quadrature channels. $P_{LR}$ was 12 ± 2% for the matching network for MEMS, PIN diode and hard‐wired configurations of the coil, which was verified with three repeated measurements.

### 3.2 Transmit uniformity and efficiency with switching

**Figure 3:**
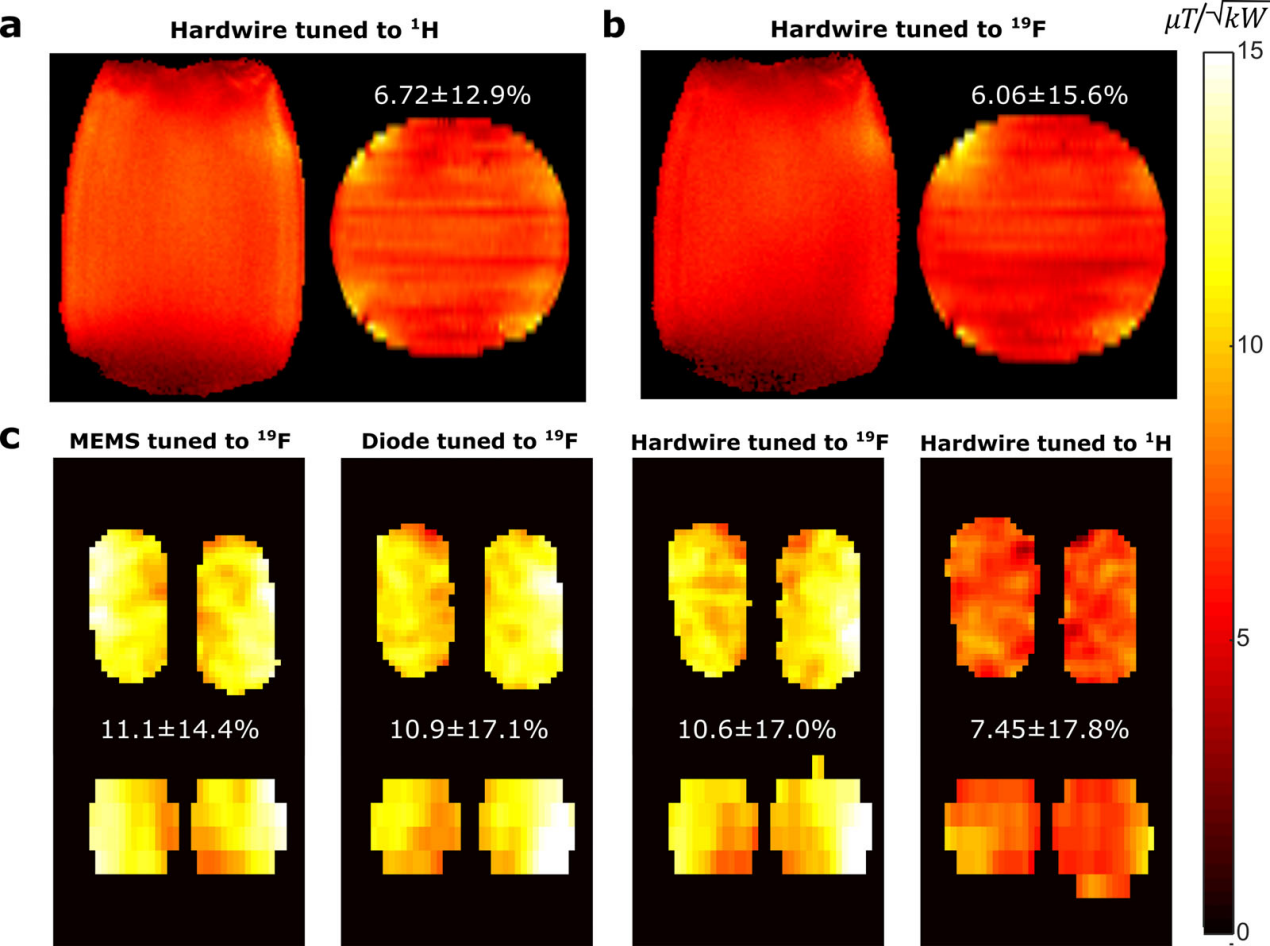
Measured transmit efficiency at 63.8 MHz within a cylindrical phantom in central axial and coronal slices. In measurement, the coil is either tuned to ^1^H (**a**) or ^19^F (**b**) frequency. The mean transmit efficiency shown above axial slices is calculated within the volume of the circled region. (**c**) Measured transmit efficiency within multi‐nuclear phantom tuned to ^19^F frequency using the three methods: MEMS switch, PIN diode, hard‐wired connection and additionally the coil tuned to ^1^H frequency

The measured T_1_ of ^1^H in the salt solution phantom was 39.5 ms, while the T_1_ of ^19^F in the C_3_F_8_/O_2_ mixture was 16.6 ms. Using the FA mapping method described, the measured transmit efficiency within a cylindrical phantom at 63.8 MHz is shown in Figure 3A and Figure 3B, when the coil is hard‐wired tuned to ^1^H and ^19^F respectively. A measured reduction of ∼12% in the mean transmit efficiency and a ∼21% increase in the B_1_ inhomogeneity (SD) was observed when the resonance of coil was set to the ^19^F frequency, while transmitting and receiving at the ^1^H frequency. Similarly, for ^19^F, the mean transmit efficiency decreased by 32% and the B_1_ inhomogeneity (SD) increased by 67% when the resonance of coil was set to ^1^H.

The measured transmit efficiency for the three switching methods are presented in Figure 3C. The mean and SD of the transmit efficiency calculated from the fitted FA from Eq. ((1)) is displayed above the axial images. The ρ/σ within the ^19^F phantom, as fitted from Eq. ((1)), was 43.6 ± 17.8% with MEMS, 43.4 ± 23.1% with PIN diode, 48.7 ± 28.1% with hard‐wired ^19^F tuning and 44.6 ± 28.6% with hard‐wired ^1^H tuning. The mean SNR changed marginally when tuning was switched from ^19^F to ^1^H, but the SD increases demonstrating a reduction in homogeneity of the transmit and receive sensitivity profiles. Results of transmit efficiency and SAR simulation^21^ for a cylindrical phantom and realistic human body model with HFSS^®^ (ANSYS, Canonsburg, PA) and Sim4Life^®^ (duke model)^22^ (ZMT, Zurich Switzerland) can be viewed in the Supporting Information Figure S2, which substantiate the measured results.

### 3.3 In vivo imaging

**Figure 4:**
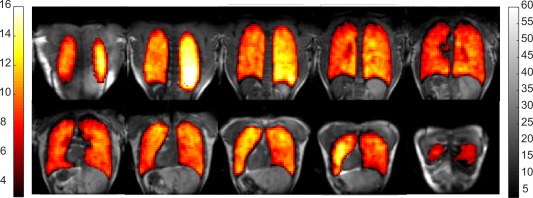
Coronal ^19^F ventilation images overlaid upon ^1^H images from a healthy volunteer (male, 28 years old) using MEMS to switch the coil resonance.

Eight central slices of ^19^F in vivo lung ventilation images overlaid on ^1^H images are shown in Figure 4. The resulting inhaled ^19^F C_3_F_8_ lung ventilation MRI display similar SNR homogeneity to those performed with the phantom. SNR was found to be high enough ( ∼12) with the given imaging parameters for single breath‐hold lung ventilation images to be obtained and co‐registered with proton structural images.

## 4 DISCUSSION

In this study, there was no measurable difference in the power loss introduced by MEMS or PIN diode switching ( $P_{LR}$) when compared with a hard‐wired connection, as would be expected from their relatively low nominal ESR (Supporting Table S1). The loss in the matching networks is less than the insertion loss incurred across the power‐dividers used to feed power to the coil elements (0.3–0.5 dB loss for each stage) and primarily comes from the use of inductors, which have physically limited Q factors. There was only a 4.5% difference (10.6–11.1 $\mu T/\sqrt{\text{kW}}$) in the mean transmit efficiency measured with the three switching configurations, which is likely in part due to the variation in re‐positioning the phantom. This is in accordance with other studies, which showed similar imaging performance with switched dual‐tuned coils when compared with single‐tuned counterparts.^12^, ^23^ Therefore, we believe the choice of switching method is primarily one of practicality and we summarize below the salient considerations.

From a component perspective, MEMS typically have a higher cost and occupy a larger circuit footprint than PIN diodes. PIN diodes require high DC power consumption and biasing requires multiple inductive chokes to prevent RF currents induced on DC lines, rather than resistive networks. MEMS switches typically require higher DC voltages, because their operation is based on electrostatic actuation, which would require the scanner interface to be in accord with voltage directives for medical devices.^24^ However, to prevent unintentional forward biasing of PIN diodes in their off‐state requires higher reverse bias voltage.^25^ Additionally, without sufficient reverse biasing the isolation is degraded and the transmitted power becomes nonlinear,^26^ which can lead to device destruction,^27^ as demonstrated here with high power pulse leading to diode burnout. The lower switching speed of MEMS switches when compared with PIN diodes is listed in Supporting Table S1, but previous research has demonstrated MEMS switches have adequate switching speed for most MR imaging methods, and are comparable to that of PIN diodes including driver circuitry.^7^, ^28^, ^29^

In this study, matching the coil to the correct frequency reduced the reflection coefficient from ∼‐5 dB to < ‐20 dB, which corresponds to an increased mean transmit efficiency and homogeneity at the ^19^F and ^1^H frequency. Therefore, a clear advantage of the use of dual‐tuning was identified, despite the relatively close frequencies. Nevertheless, in situations where the required scope of ^1^H imaging is limited, e.g., for initial localizer/survey/pilot imaging or low‐resolution structural lung imaging in the same‐breath, a coil optimized for ^19^F frequency could be sufficient for ^1^H imaging. The limitations of using the coil in this manner depends on the loaded quality factor of the coil, which primarily depends on the physical dimension of individual element/loop.

Although the primary theme of the work was the switching comparison, the ^19^F perfluoropropane lung image quality obtained with the transceiver coil at 1.5 T is encouraging, as 1.5 T may have potential benefits over 3 T for this application in terms of reduced SAR and longer $T_{2}^{*}$ of the gases in vivo.

## 5 CONCLUSIONS

The losses introduced by switching a dual‐tuned coil between ^19^F and ^1^H with either MEMS or PIN diode switches was found to be not measurably different to the losses experienced with hard‐wired connections. Moreover, the MEMS switch did not fail during high RF power pulsing. Therefore, we believe MEMS switches are suitable for use in high power transmit coils and may be used in applications, which currently use PIN diodes or in T‐R switch networks for dual tuned MRI coils.

## Supporting information


**FIGURE S1** Measured reflection coefficient (for right coil element) when coil tuned to ^1^H or switched to ^19^F tuning by the three methods: MEMS, PIN Diodes and hard‐wired
**FIGURE S2** (A) Simulated transmit efficiency at 63.8 MHz within a cylindrical phantom in central axial and coronal slices. Simulated transmit efficiency (µT/$\sqrt{kW}$) with 1 kW RMS input power at 60 MHz using HFSS (B) or SIM4LIFE (C) using realistic human body models. The mean transmit efficiency ± standard deviation shown above axial slices is calculated within the volume of the circled region with phantom and over the displayed region in human body models. Greater inhomogeneity is observed in HFSS human model due to the larger size, thereby having regions much closer to conducing elements of coil. However, Local 10g averaged SAR for the same input power calculated by HFSS or SIM4LIFE with the body models at 60MHz were close at 121 W/kg and 125 W/kg, respectively
**TABLE S1** Performance parameters of common switching devices: MEMS, PIN diodes, and FETs.
