# Supplementary material for: Comparison of MEMS switches and PIN diodes for switched dual tuned RF coils
Source: Magn Reson Med. 2018 Mar 9;80(4):1746–53. doi: 10.1002/mrm.27156 (PMC6120476; doi:10.1002/mrm.27156)
Supplement: Supplementary file 1 — FIGURE S1 Measured reflection coefficient (for right coil element) when coil tuned to 1H or switched to 19F tuning by the three methods: MEMS, PIN Diodes and hard‐wired FIGURE S2 (A) Simulated transmit efficiency at 63.8 MHz within a cylindrical phantom in central axial and coronal slices. Simulated transmit efficiency (µT/kW) with 1 kW RMS input power at 60 MHz using HFSS (B) or SIM4LIFE (C) using realistic human body models. The mean transmit efficiency ± standard deviation shown above axial slices is calculated within the volume of the circled region with phantom and over the displayed region in human body models. Greater inhomogeneity is observed in HFSS human model due to the larger size, thereby having regions much closer to conducing elements of coil. However, Local 10g averaged SAR for the same input power calculated by HFSS or SIM4LIFE with the body models at 60MHz were close at 121 W/kg and 125 W/kg, respectively TABLE S1 Performance parameters of common switching devices: MEMS, PIN diodes, and FETs. [file MRM-80-1746-s001.docx]

Supplementary Table S1: Performance parameters of common switching devices: MEMS, PIN diodes and FETs.

| **Device** | **Isolation**  **Impedance** | **Switching Speed (µs)** | **Current**  **(mA)** | **Cost** | **Size** | **Control**  **Voltage** | **Peak Current** | **Stand-off**  **Voltage** | **R_on_**  **Ω** |
| --- | --- | --- | --- | --- | --- | --- | --- | --- | --- |
| **PIN Diode**  MA4P7435F-1091T | < 3 pF | 0.35-35 (6, 25, 29) | > 100 | low | small | $\sim$5V | 10 A | Peak Reverse  1100V | < 0.3 |
| **GaAs FETs** | 3.1-7.1 pF  (6) | Similar to diode (6) | < 0.001 | medium | medium | $\sim$5V | 2-6.3 A  (6) | Vds breakdown  $\sim$100 V (6) | 0.28-0.6  (6) |
| **MEMS**  **MM7100** | < 2 pF | $\sim$4 | < 0.001 | high | large | 82 V | 5 A | 500 V | $\sim$0.4 |


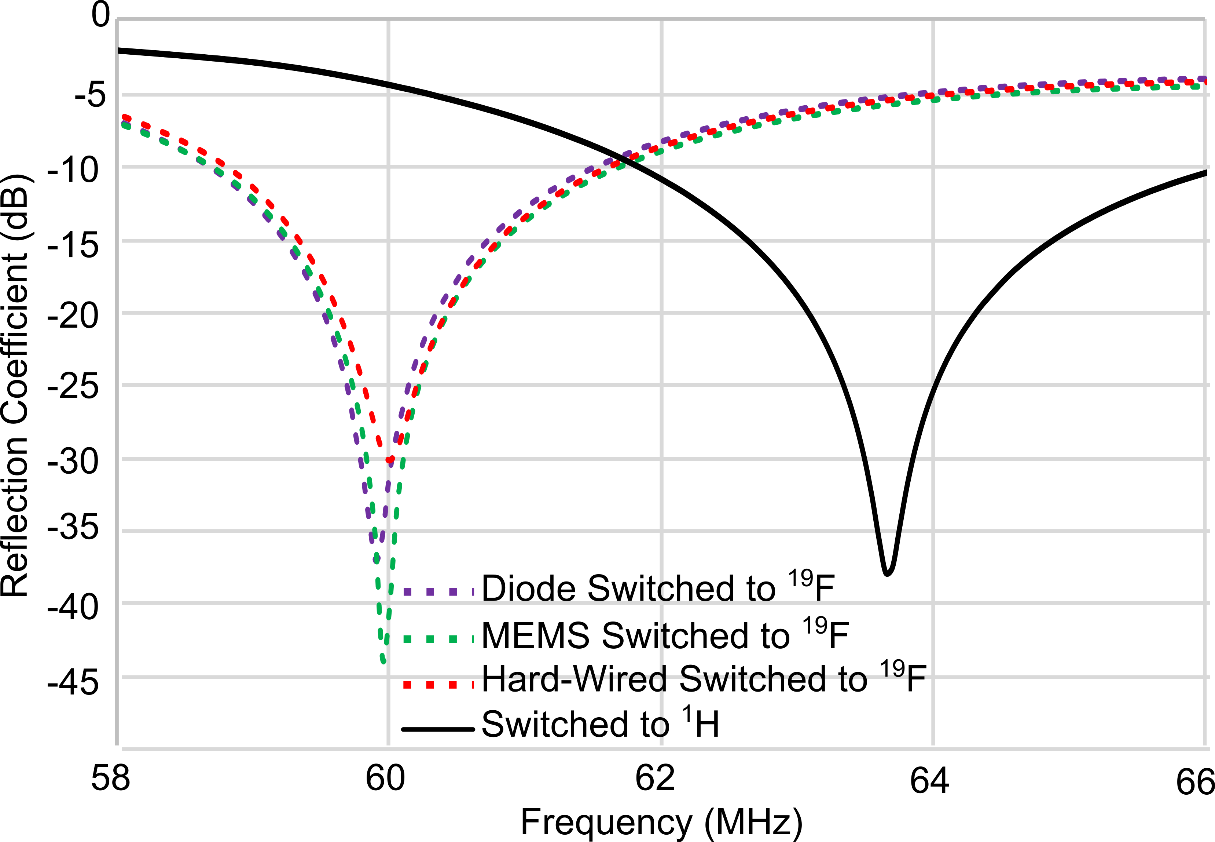


Supplementary Figure S1: Measured reflection coefficient (for right coil element) when coil tuned to ^1^H or switched to ^19^F tuning by the three methods: MEMS, PIN Diodes and hard-wired.


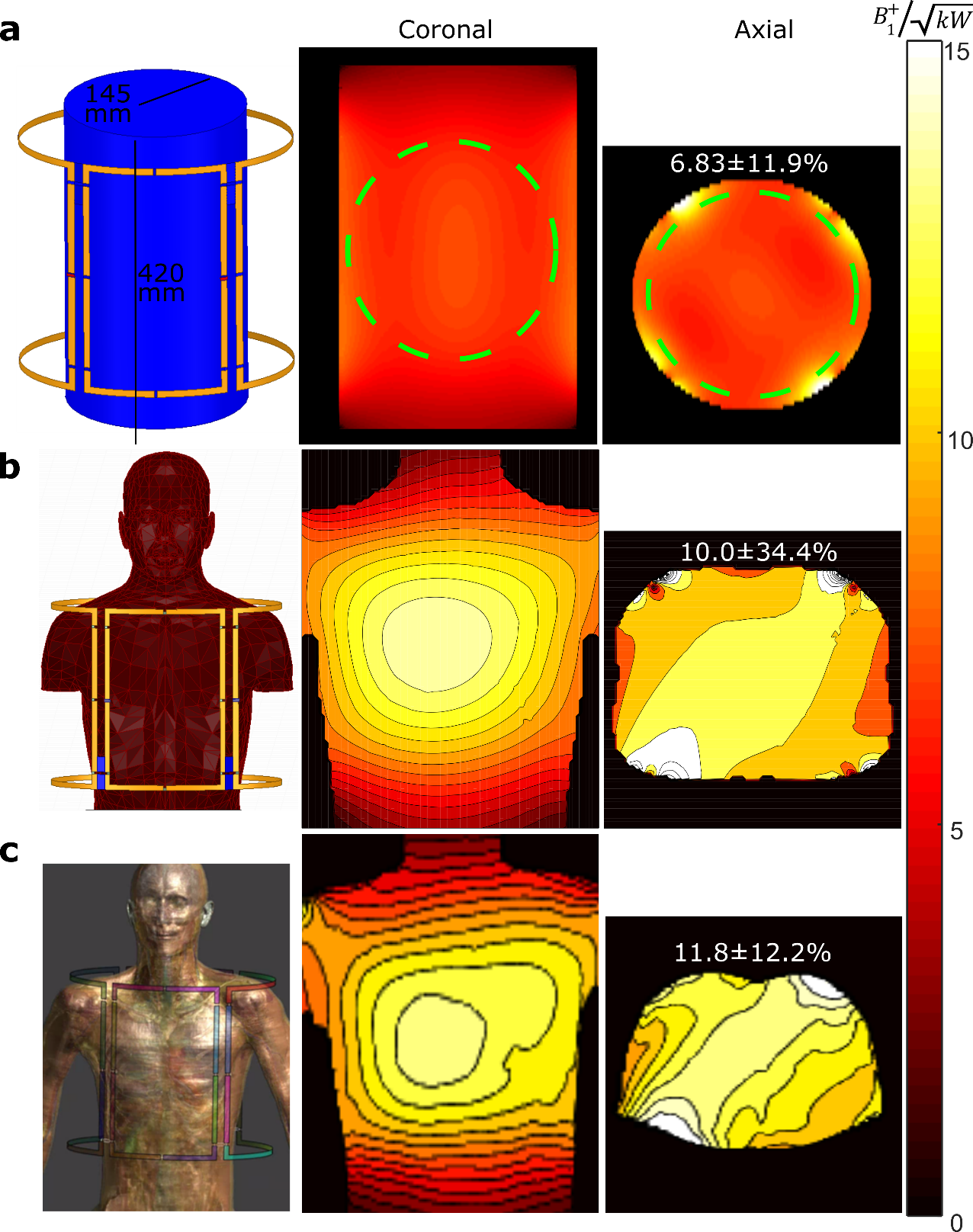


Supplementary Figure S2: **a:** Simulated transmit efficiency at 63.8 MHz within a cylindrical phantom in central axial and coronal slices. Simulated transmit efficiency (µT/$\sqrt{kW}$) with 1 kW RMS input power at 60 MHz using **b:** HFSS or **c:** SIM4LIFE using realistic human body models. The mean transmit efficiency ± standard deviation shown above axial slices is calculated within the volume of the circled region with phantom and over the displayed region in human body models. Greater inhomogeneity is observed in HFSS human model due to the larger size, thereby having regions much closer to conducing elements of coil. However, Local 10g averaged SAR for the same input power calculated by HFSS or SIM4LIFE with the body models at 60MHz were close at 121 W/kg and 125 W/kg, respectively.
